# Integrative Analyses of Transcriptomics and Metabolomics in Sex Differentiation of Mulberry Flowers

**DOI:** 10.3389/fmolb.2022.881090

**Published:** 2022-05-05

**Authors:** Pei-Gang Liu, Zi-Long Xu, Yan Zhu, Tian-Bao Lin, Zhi-Qiang Lv, Sheng Yang, Jin-Wang Wang, Wen-Jun Hu, Lin Chen, Jia Wei

**Affiliations:** ^1^ Institute of Sericulture and Tea, Zhejiang Academy of Agricultural Sciences, Hangzhou, China; ^2^ Institute of Subtropical Crops, Zhejiang Academy of Agricultural Sciences, Wenzhou, China

**Keywords:** transcriptomics, metabolomics, sex determination, sex differentiation, mulberry flowers

## Abstract

Sex determination and sex differentiation of plants are important physiological processes of plant development. Mulberry (*Morus indica* L.) is an important economic tree being cultivated in sericulture countries, and mulberry leaf is commonly used for sericulture. The transcriptomic and metabolomic differences between the staminate flowers (SFs) and pistillate flowers (PFs) of mulberry were investigated by RNA sequencing and ultra-performance liquid chromatography-tandem mass spectrometry (UPLC-MS/MS). Overall, we uncovered 4,230 genes and 209 metabolites are significantly differentially expressed between the SFs and PFs of mulberry. The combined transcriptomic and metabolomic analysis revealed these differentially expressed genes (DEGs) and differentially expressed metabolites (DEMs) are involved in flavonoid biosynthesis, galactose metabolism, plant–pathogen interaction, and starch and sucrose metabolism, and these detected DEGs and DEMs may be associated with sex differentiation of mulberry through the regulation of the enrichment pathways, such as the MAPK pathway, flavonoid biosynthesis, galactose metabolism, plant–pathogen interaction, and starch and sucrose metabolism. This study will provide a rich source for the analysis of the molecular mechanism of mulberry sex differentiation processes.

## Introduction

Mulberry (*Morus indica* L.) is a deciduous woody tree that has significant economic value, being a plant with a long history of application in sericulture. For more than 5,000 years, the mulberry leaf has served as an extremely valuable commercial source of food for silkworm, and it has made a significant contribution to humans (Jia et al., 2014; Khamenei-Tabrizi et al., 2020). In addition to using its fused leaves as food for silkworms, differential organs of the mulberry tree have been wildly used as traditional herbal medicines, such as SANG YE (*Folium Mori*), SANG BAI PI (*Cortex Mori*), and SANG ZHI (*Ramulus Mori*) (Yuan and Zhao, 2017; He et al., 2018). The mulberry fruits (*Fructus Mori*) are delicious and nutritious, having been scientifically proven to possess beneficial health effects for people (Dong et al., 2021). Presently, under the impact of the modern textile industry, traditional sericulture is on the decline, and diversified development has become an important path for its transformation and upgrading (Buhroo et al., 2018). More and more nutritional products of mulberry are appearing in modern people’s life, such as mulberry tea, mulberry wine, mulberry vinegar, and mulberry jam (Dhiman et al., 2019; Rohela et al., 2020). Furthermore, because of its good absorption abilities for both dust and heavy metals, along with a high tolerance of salinity, mulberry trees are increasingly being used for soil improvement and environmental remediation projects (Lei et al., 2018; Jiang et al., 2019).

One key aspect of sericulture’s diversification is the breeding of new special mulberry varieties with suitable plant traits. For mulberry, cross pollination remains the most promising technique of plant breeding to create a better cultivar with desirable traits. Accordingly, there are over four thousand varieties of mulberry distributed worldwide, which have different biological characteristics and pharmacodynamics values (He et al., 2013; Yu et al., 2018). Mulberry flower is monecious or diecious in nature, and cross pollination of mulberry will inevitably be affected by some monecious mulberry varieties. Hence, clearly understanding the mechanisms of sex determination and sex differentiation at the molecular level will help in breeding robust mulberry cultivars with desired traits.

The development of floral sex determination and sex differentiation in plants is a complex process involving interplay between genetic determinants, the environment, and hormones (Dellapora and Calderon-Urrea, 1994). Yet surprisingly, few studies have report that attempted to develop sex identification of mulberry. Thomas (2004) has demonstrated that sex expression in mulberry could be modified *in vitro* by ethrel and silver nitrate. Genes participating in floral bud development and male bulk-specific RAD tags of mulberry were identified; these genes may play an important role in flowers’ sex determination and sex differentiation of mulberry, and they could be used as dominant genetic markers for the dissection of sex determination and sex differentiation in mulberry (Shang et al., 2017; Atsumi et al., 2019). In addition to genetic regulation, sex determination and sex differentiation processes have also been reported to be regulated by affecting metabolomic processes in some plants (Liao et al., 2020; Jiang et al., 2021); however, till now, there was no report on the metabolomic differences related to sex determination and sex differentiation of mulberry. Based on these findings, it is very interesting to study the differences in physiological traits associated with sex determination and sex differentiation of mulberry using multiomics techniques.

In this study, the gene expression and metabolomics between the staminate flowers (SFs) and pistillate flowers (PFs) of mulberry were compared by integrated transcriptome and metabolome analysis. The results will provide an important theoretical basis for revealing the molecular mechanism of mulberry sex differentiation system regulation, and they may be useful for exercising better control over the development of mulberry flower.

## Materials and Methods

### Plant Materials

The samples of SFs and PFs were collected from Qiangsang 3, a monecious mulberry variety currently cultivated at the resource nursery of the Mulberry Research Section, Zhejiang Academy of Agricultural Sciences located in Hangzhou, Zhejiang Province, China (120.20°E, 30.32°N). On 19 March 2020, 30 SFs and PFs under a similar development schedule (i.e., day two of flowering) from three mulberry trees (ten per tree) were sampled, respectively, and pooled together as one biological replicate, in this way, each flower sample includes three sample replicates from nine differential mulberry trees.

After harvesting, all six mulberry flower samples were then immediately frozen in liquid nitrogen and stored in polyethylene bags at −80°C until their RNA and metabolite extraction.

### Transcriptomic Analysis

The total RNA of the SFs and PFs of mulberry was extracted from their ground frozen pellets using the TransZol Plant RNA Kit (Transgen, Beijing, China). Paired-end cDNA sequencing libraries were prepared with the IlluminaTruSeq Stranded Total RNA Library Preparation Kit following the manufacturer’s protocol. The RNA was quantified using the Qubit RNA Assay Kit in Qubit 2.0 Fluorometer (Life Technologies, Carlsbad, CA, United States), after which the integrity of each RNA sample was checked with the RNA Nano 6000 Assay Kit of the Agilent Bioanalyzer 2,100 system (Agilent Technologies, Santa Clara, CA, United States). The final cDNA libraries were sequenced on the Illumina NovaSeq 6,000 platform by Wuhan Metware Biotechnology Co., Ltd., Wuhan, China. The mulberry reference genome and corresponding gene annotation files were downloaded directly from the mulberry genome website (http://morus.swu.edu.cn/morusdb).

### Gene Functional Annotation and Expression Level Analysis

To obtain high-quality Illumina sequencing data consisting of clean reads, the raw reads were first assessed and then processed to remove any Illumina adapters, unknown nucleotides (i.e., those more than 10% of N), and low-quality reads (those more than 50% of Q ≤ 20 bases). Next, the assembled genes were annotated using BLASTX searches against the databases, mainly the NR protein database and COG database. Additional annotations were obtained from the Kyoto Encyclopedia of Genes and Genomes (KEGG) gene and protein family database through the KEGG Automatic Annotation Server (KAAS). The genes exhibiting differential expression between the SFs and PFs were then identified using the number of mapped reads as edge R inputs (http://www.bioconduct or. org/packages/release/bioc/html/dege R. html). The genes that underwent a change of two-fold or more between samples of the two flowers and had a false discovery rate (FDR) of 5% or less, were designated as DEGs (differentially expressed genes).

### Metabolite Extraction and UPLC-MS Sample Preparation

The mulberry flower samples were first freeze-dried by using a vacuum freeze dryer (Scientz-100F), and then, each sample was homogenized with zirconia beads in a mixer mill (MM 400, Retsch) at 30 Hz, for 1.5 min. The ensuing powdered samples (100 mg) were extracted using 0.6 ml of 70% aqueous methanol at 4°C overnight. The samples were each vortexed six times during this extraction. After their centrifugation at 10,000 × g for 10 min, the supernatant was transferred to a new centrifuge tube and filtrated through a 0.22 μm pore size filter system (SCAA-104) before conducting the UPLC-MS/MS analysis.

### Metabolite Detection by UPLC-MS/MS

The chromatographic separations were performed on a Waters ACQUITY UPLC HSS T3 system (Shim-pack UFLC SHIMADZU CBM30A, www.shimadzu.com.cn/), equipped with a C18 column (1.8 μm × 2.1 mm × 100 mm) maintained at 40°C. A mobile phase system of A (0.1% formic) and B (acetonitrile) was used for the elution. The gradient program was expressed as follows: ratio of A: B of 95:5 (v/v) at 0 min, then 5:95 (v/v) at 11.0 min, 5:95 (v/v) at 12.0 min, and 95:5 (v/v) at 12.1 min, ending with 95:5 (v/v) at 15.0 min. The flow rate was set to 0.35 ml/min and the injection volume was 4 μl. A quality control (QC) sample was prepared by mixing the sample extracts; it was injected regularly, after every set of 10 samples, to monitor any changes in the repeated analysis.

A triple quadrupole-linear ion trap mass spectrometer (Applied Biosystems 6500 Q TRAP, www.appliedbiosystems.com.cn/) equipped with an ESI Turbo Ion-Spray was employed to analyze the metabolites. The ESI source operation parameters were as follows: an ion source, turbo spray; source temperature, 500°C; ion-spray voltage (IS), 5500 V; ion source gas I (GSI), gas II (GSII), and curtain gas (CUR) of 55, 60, and 25 psi, respectively; and high collision gas (CAD). Metabolite quantification was carried out using the multiple reaction monitoring models as previously described (Chen et al., 2013; Yuan et al., 2018). The metabolites were identified using the self-built database MWDB (Met Ware database, Wuhan MetWare Biotechnology, Wuhan, China) and KNAPSAcK (http://kanaya.naist.jp/KNApSAcK/), MassBank (http://www.massbank.jp/), MoToDB (http://www.ab.wur.nl/moto/), HMDB (http://www.hmdb.ca/), and METLIN (http://metlin.scripps.edu/index.php) public databases of metabolite information, following the standard operating procedures.

The metabolites with significant differences in content were determined according to the following criteria: variable importance in the projection value ≥ 1 and fold change ≥ 2 or ≤ 0.5.

### Quantitative Reverse Transcription PCR Analyses of DEGs

A portion of the pooled total RNA used for the RNA-Seq analysis was used to verify the results by quantitative reverse transcription PCR (qRT-PCR), and ten genes potentially involved in sex differentiation were randomly selected for the expression patterns analysis. The primers for qRT-PCR were designed using Primer3 software version 0.4.0 (http://frodo.wi.mit.edu/primer3/), and were listed in Supplementary Table S1. Three biological replicates for each sample were performed on a Bio-Rad iQ5 Optical System Real-Time PCR System (Bio-Rad, USA) using an SYBR Green-based PCR assay. Its thermal program went as follows: 95°C for 3 min followed by 40 cycles of 30 s at 95°C, 20 s at 58°C, and 15 s at 72°C. The relative genes expression levels of each sample of mulberry flower type were quantified using the 2^−△△CT^ method, for which the ribosomal protein gene of mulberry served as the internal reference gene (Livak and Scmittgen, 2000; Shang et al., 2017).

## Results

### Phenotype of Staminate Flowers and Pistillate Flowers

**FIGURE 1:**
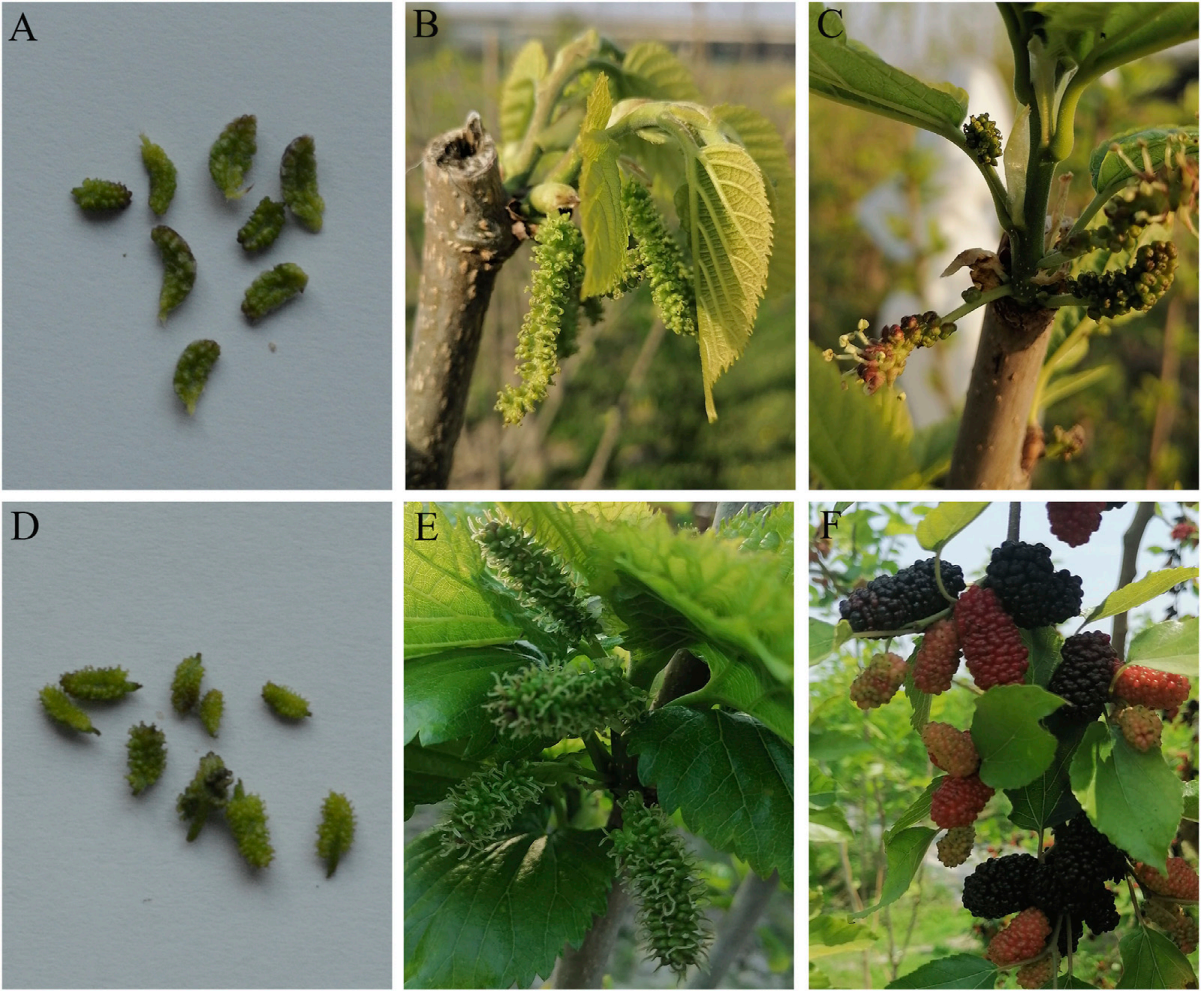
Phenotypes of the staminate flowers (SFs) and pistillate flowers (PFs) of mulberry at differential development stages. **(A)** SFs of mulberry at the early flowering stage. **(B)** Expanded SFs with stamens. **(C)** Mature SFs whose anthers naturally crack and pollen have wind-aided dispersal. **(D)** PFs of mulberry at the early flowering stage. **(E)** Expanded PFs with stigma waiting for pollination will become small fruits. **(F)** Red or purple fruits.

Mulberry flowers were picked out from the mulberry buds from March to April, and the flower was composed of many small flowers clustered around the flower axis forming a spike-like arrangement. Qiangsang 3 is a monecious mulberry variety that produces staminate flower (SFs, Figure 1A) and pistillate flower (PFs, Figure 1D) on one branch.

The SFs (i.e., a flower cluster) of mulberry consists of many small flowers, each containing four stamens, while the PFs flower cluster has many pistillate stigmas with a small paintbrush. SFs will expand (Figure 1B) and their anthers will naturally crack after maturation and pollen wind-aided dispersal and spread to the stigma of PFs (Figure 1C). After fertilization, the stigma of PFs will wither, and the ovary wall and perianth gradually expand to form mulberry fruits (Figure 1E), whose color starts to turn red, and eventually purple as they ripen (Figure 1F).

### Identification of DEGs Between SFs and PFs of Mulberry

Based on a fold change > 2 and a corrected *p* value <0.05, overall, 4,230 genes were found to be significantly differentially expressed between the SFs and PFs of mulberry. Among these DEGs, 1832 were upregulated and 2,398 were downregulated in the SFs with respect to the PFs; all this DEGs information is summarized in Supplementary Table S2. These results suggested the DEGs between SFs and PFs play important roles in the sex differentiation of mulberry flowers.

### GO and KEGG Analysis of DEGs Between the SFs and PFs of Mulberry

**FIGURE 2:**
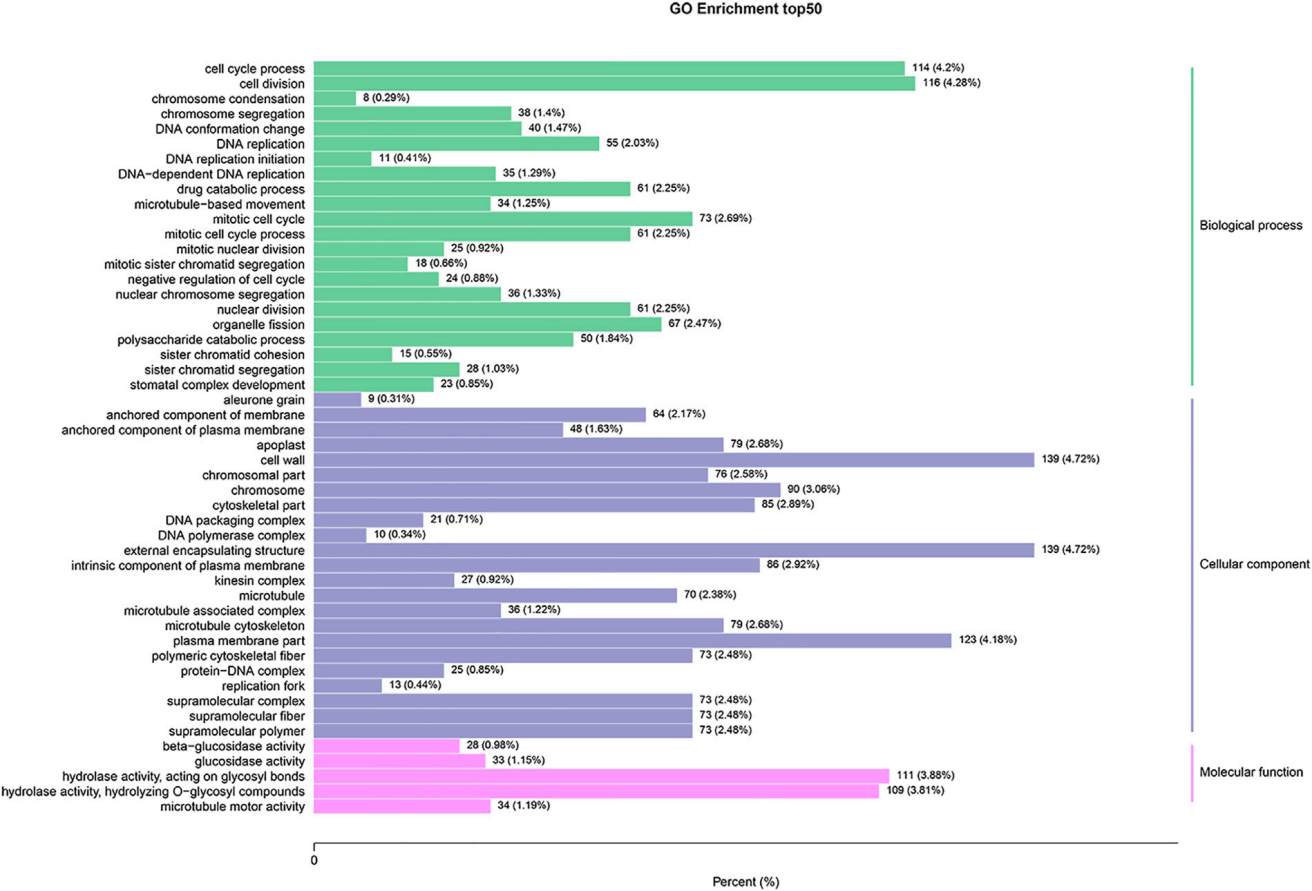
GO enrichment analysis of DEGs between SFs and PFs. The results are summarized under the three top-level ontologies: biological process, molecular function, and cellular component. The left y-axis indicates the percentage of a specific GO category subsumed in one of the three main categories mentioned above. The right y-axis indicates the annotated gene number expressed in the given sub-category. The downregulated DEGs are indicated in blue, and the upregulated DEGs are in red.

To better understand the biological functions of the identified DEGs between SFs and PFs of mulberry, they were functionally classified using GO enrichment analysis. Of these 4,230 DEGs, 2,911 (68.8%) had annotations to 3,037 GO terms, whose data are summarized in Supplementary Table S3. The DEGs were significantly enriched in 70 GO terms, and the top 20 enrichment GO terms appear in Figure 2. The DEGs are mainly significantly enriched in the categories of cellular component (CC, 27 terms) and the biological process (BP, 33 terms).

Of the 10 terms in the molecular function (MF) category, “hydrolase activity, hydrolyzing O-glycosyl compounds,” and “hydrolase activity, acting on glycosyl bonds” were two terms that were more significantly enriched, both being related to hydrolase activity. The most abundant GO terms under BP were cell cycle process, cell division, mitotic cell cycle, and organelle fission. In the CC category, cell wall, external encapsulating structure, plasma membrane, and chromosome were the most abundant GO terms uncovered.

**FIGURE 3:**
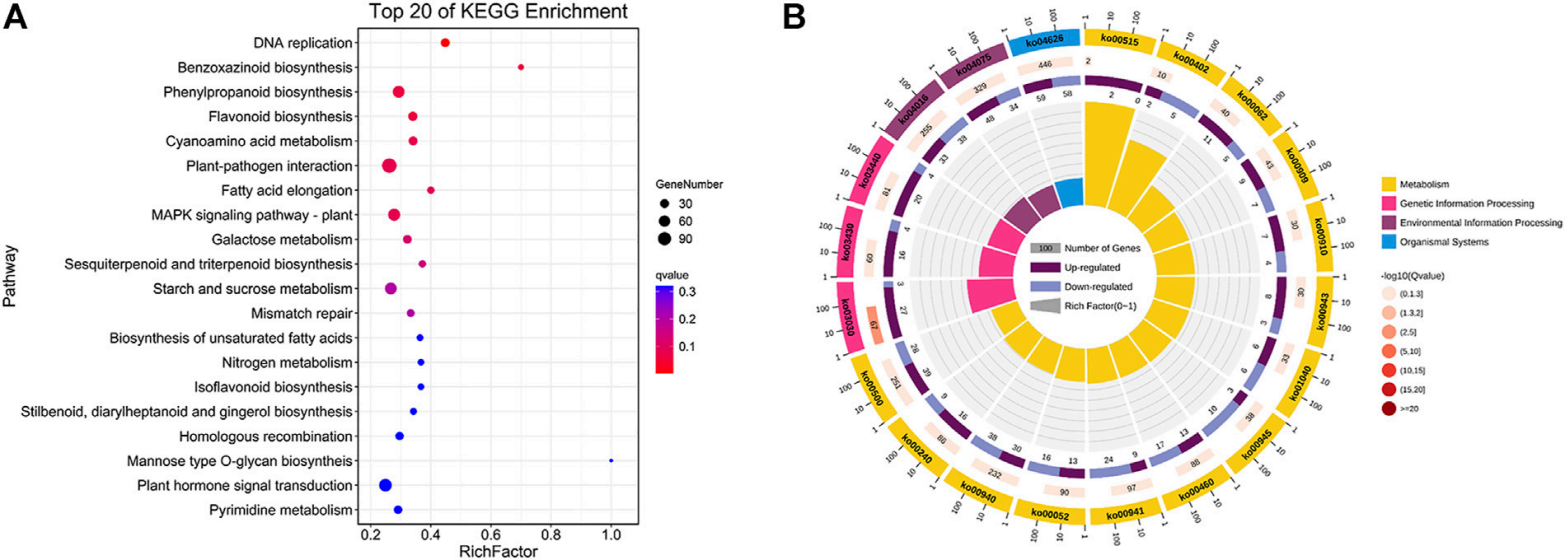
KEGG enrichment analysis of DEGs between the SFs and PFs of mulberry. **(A)** The color of the balls indicates the *p* value of the KEGG enrichment analysis. Red indicates the highest *p* value and blue the lowest (least likely to occur by chance), and the size of the ball represents the number of DEGs enrichment in the pathway. **(B)** Top 20 enrichment pathways of DEGs are categorized in KEGG classification with differential colors.

To further investigate the potential functions of those DEGs between flower types, KEGG pathway enrichment analyses were performed on DEGs. In total, 1,511 DEGs were assigned to 136 KEGG pathways and these results are described in Supplementary Table S4, and the top 20 enrichment pathways are visualized in Figure 3A. From Figure 3B, the top 20 enrichment pathways are categorized in 4 KEGG classifications, such as metabolism, genetic information processing, environmental information processing, and organismal systems, and most of the top 20 enrichment pathways are categorized in metabolism classification.

According to a *p* value ≤ 0.05 that denotes significance, 17 KEGG pathways were found significantly enriched between SFs and PFs, such as those of DNA replication (ko03030), MAPK signaling pathway–plant (ko04016), flavonoid biosynthesis (ko00941), benzoxazinoid biosynthesis (ko00402), fatty acid elongation (ko00062), galactose metabolism (ko00052), cyanoamino acid metabolism (ko00460), plant-pathogen interaction (ko04626), and starch and sucrose metabolism (ko00500).

### Validation of Gene Expression Using qRT-qPCR

**FIGURE 4:**
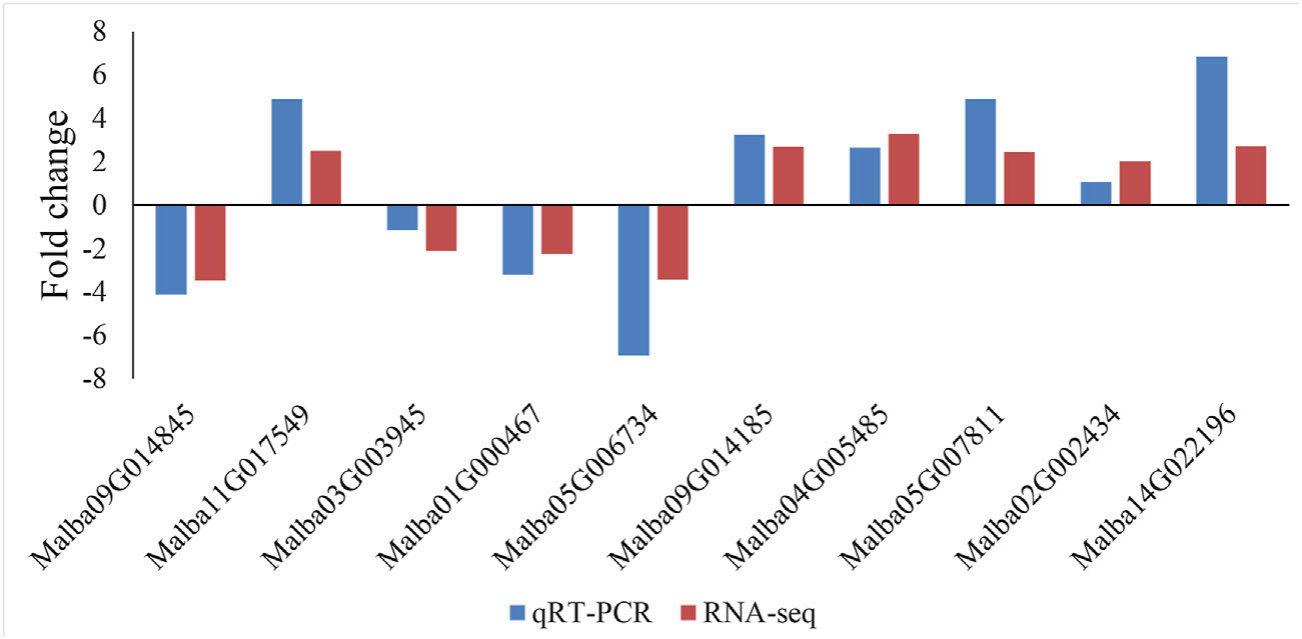
Validation of the ten chosen DEGs between the SFs and PFs of mulberry by qRT-PCR.

To confirm the expression patterns of a subset of DEGs identified by Illumina sequencing, ten genes were randomly selected for qRT-qPCR analysis (Figure 4). The RT-PCR results showed that all ten selected genes were differentially expressed between the SFs and PFs of mulberry (*p* < 0.05), and their expression levels were consistent with the RNA-seq results. Hence, the measured changes in gene expression as detected by RNA-seq rely on the actual transcriptome differences between the different flower libraries.

### Metabolite Differences Between the SFs and PFs of Mulberry

**FIGURE 5:**
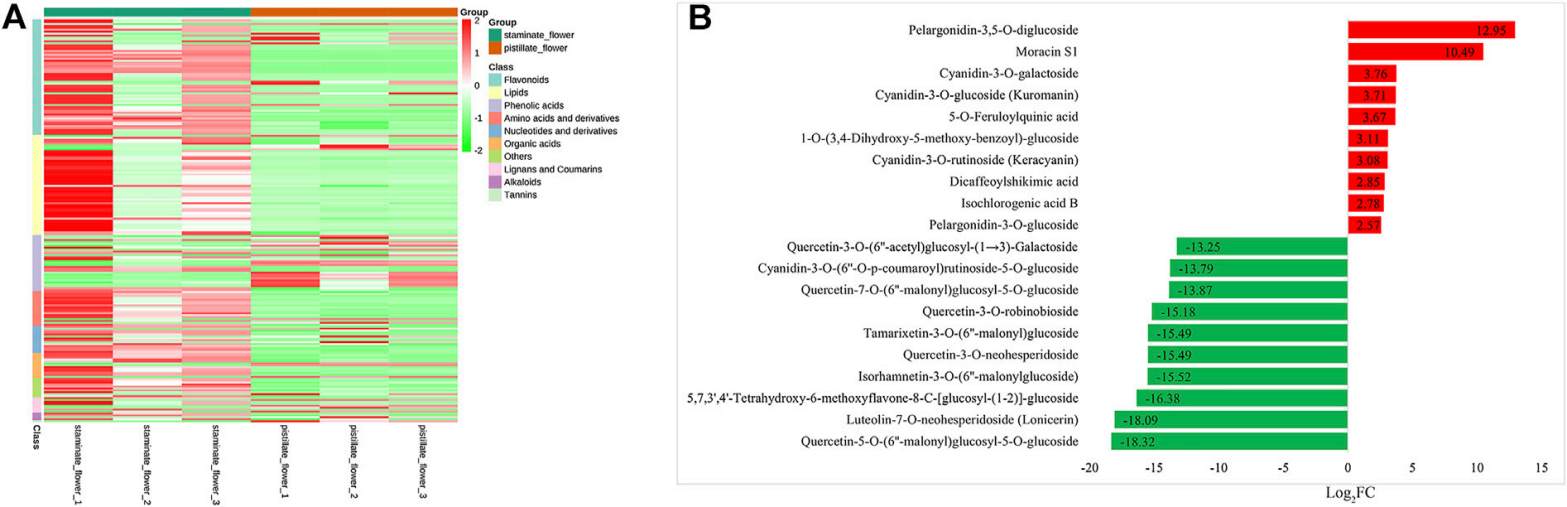
DEMs between the SFs and PFs of mulberry. **(A)** Heatmap of DEMs between the SFs and PFs of mulberry. The colors from green to red indicate the relative contents of metabolites in the SFs compared with those in the PFs. **(B)** Bar chart of top 10 DEMs of SFs and PFs, respectively. Red and green bars on the Y-axis represent up-expressed and down-expressed metabolites in SFs, respectively.

To investigate the metabolic differences between the SFs and PFs of mulberry, we created the profile of the primary metabolites using UPLC-MS/MS, and a total of 403 metabolites were obtained from the samples of the two flower types. Among these detected metabolites, 209 metabolites underwent significant changes between the SFs and PFs, in that 46 and 163 were, respectively, upregulated and downregulated in the SFs of mulberry. The details of the DEMs are provided in Supplementary Table S5. As Figure 5A shows, it can be concluded that the DEMs between SFs and PFs mainly include flavonoids, lipids, phenolic acids, and derivatives.

Among the obtained DEMs, pelargonidin-3,5-O-diglucoside (pme1793), moracin S1 (pmp000798), cyanidin-3-O-galactoside (pmf0027), cyanidin-3-O-glucoside (kuromanin) (pmb0550), and 5-O-feruloylquinic acid (lmgn003073) were expressed at higher levels in SFs of mulberry, while quercetin-5-O-(6''-malonyl) glucosyl-5-O-glucoside (pmp0706), luteo-lin-7-O-neohesperidoside (lonicerin) (pmp001709), 5,7,3',4'-tetrahydroxy-6-methoxyflavone-8-C-[glucosyl-(1-2)]-glucoside(lmnp002448), isorhamnetin-3-O-(6''-malonylglucoside) (HJAP064) were all expressed more in PFs of mulberry (Figure 5B).

### KEGG Analysis of Differential Metabolites

**FIGURE 6:**
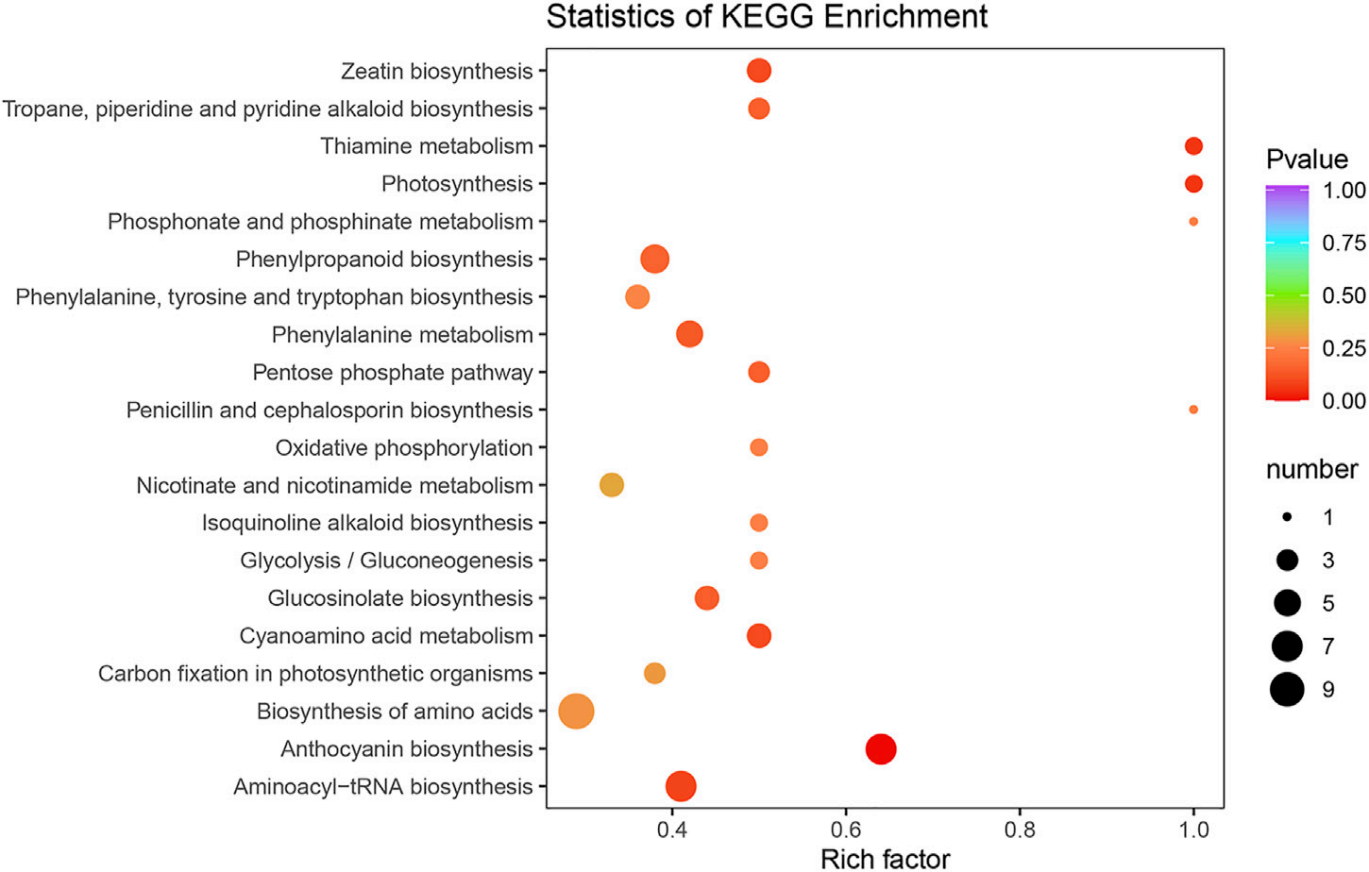
KEGG classification of DEMs between the SFs and PFs of mulberry. The color of the balls indicates the *p* value of the KEGG enrichment analysis, red indicates the highest *p* value and blue the lowest (least likely to occur by chance), and the size of the ball represents the number of metabolites enriched in the pathway.

To characterize the complex biological behaviors of the DEMs between the SFs and PFs of mulberry, the DEMs were annotated and displayed by querying the KEGG database. In total, 63 DEMs were enriched in 66 pathways and the result for these enrichment pathways are described in Supplementary Table S6. The top 20 enrichment pathways are shown in Figure 6.

Among the enrichment pathways of DEMs, most of them were significantly enriched in the MAPK signaling pathway–plant, flavonoid biosynthesis, fenzoxazinoid biosynthesis, fatty acid elongation, galactose metabolism, cyanoamino acid metabolism, plant-pathogen interaction, starch and sucrose metabolism, nitrogen metabolism, biosynthesis of secondary metabolites, homologous recombination, sesquiterpenoid, and triterpenoid biosynthesis, mannose type O-glycan biosynthesis, biosynthesis of unsaturated fatty acids, and pyrimidine metabolism. Two other pathways that were enriched belonged to the replication and repair of genetic information processing, such as DNA replication and mismatch repair.

## Discussion

Sexual reproduction is a universal phenomenon for eukaryotic organisms, in which sex determination and sex differentiation are major events. Their molecular mechanisms appear to be quite complex and can vary wildly among plants, such as mulberry, an important economic tree in some parts of the world. Previous studies have confirmed that the sex expression of the mulberry flower can be modified by some plant exogenous hormones (Thomas, 2004). Several genes were found to be related to sex determination in mulberry and can be used as genetic markers relevant to sex determination (Shang et al., 2017; Atsumi et al., 2019). However, the main mechanisms of sex determination and sex differentiation in mulberry have not been deeply determined yet. In this study, the expression changes of genes and metabolites profiling between SFs and PFs of mulberry were integratively analyzed by using RNA-seq and UPLC-MS/MS technology, and 4,230 genes and 209 metabolites were found to be significantly differentially expressed between SFs and PFs of mulberry. These results suggested that these DEGs and DEMs are associated with the sex differentiation progress of mulberry.

The results of GO enrichment analysis showed that DEGs are mainly significantly enriched in the cellular component and the biological process subcategories. Some enriched GO terms were reported to be related to sex determination in some plants in previous studies. The cell division was found to be an important enrichment GO term of target genes of differential expressed miRNAs between male and female *Asparagus officinalis* (Chen et al., 2016). The hydrolase activity is the most abundant category of DEGs between ovulate strobilus and staminate strobilus of Ginkgo biloba (Du et al., 2016).

The results of the KEGG enrichment analysis of the DEGs and the DEMs showed that they were mainly enriched in the MAPK signaling pathway, flavonoid biosynthesis pathway, starch and sucrose metabolism, and zeatin (ZT) biosynthesis.

### Putative Genes Involved in Signal Transduction

It is well appreciated that the mitogen-activated protein kinase (MAPK) pathway is crucial for the regulation of many cellular processes by integrating diverse signals, and early studies had revealed that it is an important pathway for regulating sexual dimorphism of some plants (Ge et al., 2020; Liao et al., 2020). The MAPK pathway has been demonstrated as a significantly enriched pathway of differential metabolites between male and female leaves of *G. biloba* by Liao et al., (2020). Several upregulated genes of bisexual flowers in *Cucumis melo* were found to be significantly enriched in the MAPK pathway (Ge et al., 2020). In this study, five DEGs showed enrichment in the MAPK pathway, *Malba03G003945*, *Malba05G008100*, *Malba05G007146*, *Malba14G021635*, and *Malba10G015776*; these, respectively, homologous to *calmodulin 7 (CAM 7)*, *calmodulin-like protein 10 (CML10), calmodulin-like protein 29 (CML29), calmodulin-like protein 30 (CML30), and calmodulin-like protein 45 (CML45) of the calcium-binding protein (CBP)* gene family, were all upregulated in the SFs of mulberry. The CBP genes encode a class of important messengers that contribute to the regulation of numerous cellular processes. Previous studies have found that CPBs play a key role in pollen tube germination, growth, guidance, and sperm delivery by functioning as calcium-sensitive signal molecules (Hepler et al., 2012; Tian et al., 2021). The expression of *CAM 7* was upregulated during the pollen germination and pollen tube growth of Arabidopsis, implying its critical involvement in pollen germination and tube growth (Wang et al., 2008). In other work, some *CML* genes including *CML45* were indicated to act as a positive regulator of pollen tube development in Pyrus bretschneideri (Zhou et al., 2016). Moreover, some *CBP* genes were obtained from the targeted genes of sex-specific related miRNAs in andromonoecious Populus tomentosa, suggesting that these sex-specific related miRNAs regulate calcium transport in female and male flower development (Song et al., 2013). Thus, these previous observations suggest that the overexpression of *CBP* genes in the SFs of mulberry is closely related to their curtail roles in pollen development of SFs.

One DEG that we found in the mulberry flower (*Malba02G002758*) is homologous to the *ethylene-responsive transcription factor 1B (ERF1B)* gene encoding the ERF1B, a member of the ethylene-responsive transcription factor (ERF) family. ERFs play vital roles in the plant growth and in response to hormones and abiotic stresses. Several ERFs were found to be related to ethylene signaling and sex determination in some plant species, such as *ERF5*, *ERF17*, and *ERF102. ERF 1B* was upregulated in the PFs of mulberry and may participate in sex determination by regulating the ethylene signal pathway; similar results were obtained in other species such as *Coccinia grandis*, *Cucumis sativus*, and *Cucurbita moschata* (Mohanty et al., 2017; Pawełkowicz et al., 2019; Li et al., 2020). Protein phosphatase 2C (PP2C) enzymes act as a regulator of the MAPK pathway in plants. Four DEGs which show enrichment in MAPK pathway are *Malba11G017123*, *Malba09G014845*, *Malba06G009727*, and *Malba11G017798*, are homologous to *PP2C8*, *PP2C16*, *PP2C24*, and *PP2C37*, which belong to the *PP2C* family, were all upregulated in SFs of mulberry, consistent with reports of *PP2C* genes upregulated in male floral buds of *Diospyros kaki* (Li et al., 2021). In contrast, three *PP2C* genes, *PP2C 38*, *PP2C 44*, and *PP2C 51*, were found to occur predominantly in the female flowers in bisexual catkins of *Castanea henryi*, and in *G. biloba*, one gene encoding PP2C was downregulated in male organs (Du et al., 2016; Fan et al., 2017). PP2C enzymes have been identified as the key players in plant signal transduction processes. Previous studies have confirmed that PP2C enzymes show distinct tissue expression patterns and function in differential roles in differential tissues of several plant species (Miyazaki et al., 1999; Li et al., 2018).

Flavonoids are an important class of secondary metabolites of plants that fulfill a variety of functions in plants, and they will be indispensable for attaining complete male fertility in certain plant species (Ning et al., 2018; Wang et al., 2020). The pathway for flavonoid biosynthesis was another enriched pathway between the SFs and PFs in this study. Researchers have reported that genetic sex differences result in specialized flavonoid metabolism of *G. biloba* (Liao et al., 2020). In addition, the flavonoid biosynthesis pathway was found to be associated with male floral initiation of *Jatropha curcas* (Hui et al., 2017). Other work has revealed the ability of flavonoids to regulate the activities of a wide range of proteins and thereby influence the cell growth and differentiation of plants (Brunetti et al., 2013). In this study, among the DEGs enriched in the flavonoid biosynthesis pathway, three genes, *Malba02G002432*, *Malba08G012746*, and *Malba08G012766*, all homologous to *chalcone synthase 5 (CHS 5)* are downregulated and one gene *Malba11G017549* homologous to *chalcone isomerase 3 (CHI 3)* is upregulated in the PFs of mulberry. *Chalcone synthase (CHS)* is the first enzyme of the isoflavonoid biosynthetic pathway and chalcone isomerase (CHI) catalyzes the formation of a common isoflavonoid precursor (van der Meer et al., 1992). CHS and CHI are well-known for their role in male fertility in *Oryza sativa* (Wang et al., 2020). In previous studies, *CHS* has been reported to be differentially expressed between male and female individuals of *Actinidia chinensis* and *A. officinalis* (Harkess et al., 2015; Tang et al., 2017). In *Raphanus sativus*, *CHS* is inhibited in the male-sterile phenotype and indicated that it performs an important function related to pollen wall assembly and fertility in male flowers (Yang et al., 2008). Two other genes, *Malba01G001487* and *Malba01G001617*, both homologous to the *BAHD acyltransferase gene*, were over-expressed in SFs of mulberry. Previous studies have found that BAHD acyltransferases are involved in the synthesis and elaboration of a wide variety of secondary metabolites. They were found to be specifically expressed in the tapetum cells of the anthers, and play a crucial role in pollen development of *A. thaliana* (Grienenberger et al., 2009). In *O. sativa*, a cytoplasmic *BAHD acyltransferase* gene (DPW2) has been indicated to play a fundamental role in pollen development *via* the biosynthesis of key components of the anther cuticle and pollen wall (Xu et al., 2017). Two DEGs (*Malba10G015278* and *Malba08G012764*) which are, respectively, homologous to *stilbene synthase (STS)* and *stilbene synthase 4 (STS4)*, are all over-expressed in the SFs of *mulberry. STS4* was shown to be a supermale-biased expressed gene of *A. officinalis* (Harkess et al., 2015), and several studies had demonstrated that overexpressing *STS* induces a sterile phenotype of some plants species (Ageez et al., 2005; Ingrosso et al., 2011). However, the expression differences between male and female flowers (buds) in plants are yet to be reported.

### Putative Genes Involved in Carbohydrate Metabolism

Glycolysis/gluconeogenesis has played critical roles during floral development as we all know. In this study, the enriched pathway of starch and sucrose metabolism indicated that changes were obvious between the SFs and PFs of mulberry. A previous report also showed that it was also an enrichment pathway of DEGs between female and male flowers of *Spinacia oleracea* (Li et al., 2020). The starch and sucrose metabolism pathway suppresses pollen development and ultimately leads to male sterility, it is an important enrichment pathway for differentially expressed transcripts between female and male buds of *Metasequoia glyptostroboides*, and likewise in the ovulate strobilus and staminate strobilus (Zhao et al., 2015; Du et al., 2016). Among the DEGs we obtained, twelve are humongous to *glucan endo-1, 3-beta-glucosidase (GLC)* genes and nine of them are over-expressed in PFs and three of them are over-expressed in SFs, and the differential expression change of them would seem to be associated with floral sex differentiation of mulberry. GLCs were involved in cell envelope biogenesis and cell wall metabolism. A previous study found than two GLCs were upregulated in style vis-à-vis the ovary of PFs of mulberry, and these findings suggest that GLCs may be related to the structural development of PFs of mulberry (Niu et al., 2013). In other research studies, three *GLC* genes were identified as downregulated in the stamen and petal of *Crocus sativus* (Shu et al., 2020). In addition, one GLC gene was found to have decreased in a genetic male-sterile mutant (NWMS1) of *Triticum aestivum* (Li W. et al., 2019). They were indicated to be essential for normal post-meiotic tapetal development and pollen/microspore formation in *O. sativa* (Hu et al., 2010). Thus, previous studies indicated *GLC* genes may figure prominently in the pollen development of mulberry’s SFs.

Based on the KEGG enrichment analysis of the differential metabolites between the mulberry SFs and PFs, we found some metabolites enriched in ZT biosynthesis, such as N6-isopentenyladenine (IP), uridine-5'-diphosphate-D-Xylose, 5'-deoxy-5'- (methylthio) adenosine, and adenosine 5'-diphosphate, and all of them are expressed at lower levels in the SFs of mulberry. ZT biosynthesis differed between the male and female floral buds of *D. kaki* and likewise the male and the female flowers in *S. oleracea* (Li J. et al., 2019; Li et al., 2020). ZT and its derivatives are the most important group of isoprenoids cytokinin (CKs), which be considered to promote cell division and stimulate the initiation and growth of shoots *in vitro* (Zoppi et al., 2019). There is also evidence that CKs are involved in the male reproductive development of *Arabidopsis thaliana* (Kinoshita-Tsujimura and Kakimoto, 2011). Furthermore, high levels of ZT could promote the differentiation of male floral buds leading to female unisexual flowers in *D. kaki* by enhancing the development of pistil primordia (Sun et al., 2017; Li S. et al., 2019). Cytokinin oxidase/dehydrogenase (CKXs) catalyzes the irreversible degradation of CKs. Most CKX genes are reported to be over-expressed in the reproductive organs of *Brassica napus* (Liu et al., 2018). Three CKXs (CKXs 1, 3, 4) were found to be downregulated in the SFs of mulberry in the present study, and a similar result was obtained by Xiao et al. (2020) in which CKXs were upregulated in male flowers of *Trachycarpus fortune*.

Galactose is an important energy-providing nutrient of plants and galactose metabolism has been shown to correlate closely with some plant physiological processes, such as stress tolerance, floral development, and fruit ripening. In previous studies, galactose metabolism was indicated to be linked to pistil abortion, and pistil deletion of *Prunus mume* and *Camellia sinensis* (Shi et al., 2012; Liu et al., 2020). Also, galactose metabolism appeared to differ starkly between the male and female inflorescences of *Borassus flabellifer palm* (Pipatchartlearnwong et al., 2019). 3-ketoacyl-ACP synthase (KAS III) and *alpha-galactosidase* which participate in the galactose metabolism pathway were specifically expressed in male flowers of *Vernicia fordii* (Mao et al., 2017). In this study, among the DEGs’ enrichment in the galactose metabolism pathway, two genes (*Malba05G007849* and *Malba07G011885*) both homologous to *beta-galactosidase 3 (BGAL3)* were found to be over-expressed in the PFs, whereas *Malba07G010805* (homologous to *beta-galactosidase 11, BGAL11*) was upregulated in the SFs.while two genes (*Malba02G002434* and *Malba14G021157*) both annotated as *Acyl carrier protein 1 (ACP1)* were also upregulated in the PFs of mulberry. In previous studies, three *beta-galactosidase* genes (*βGALs*) were upregulated in the male lines of *C. sativus* (Pawełkowicz et al., 2019). In *Brassica campestris*, most of the βGALs (6/7) were found to present a downward trend in the sterile flower buds compared to fertile flower buds (Zhou et al., 2019), indicating that *βGALs* may play important roles in pollen wall synthesis and regulation of male flowers). ACP 1 belongs to the acyl carrier protein (ACP) family which functions in the synthesis of fatty acids, polyketides, and non-ribosomal peptides, yet there is surprisingly little information about its involvement in regulating the sex differentiation of plants (Perez et al., 2015). Further studies are needed to determine the effects of *ACP* genes on the sexual development of SFs and PFs of mulberry.

### Transcription Factors From DEGs

Transcription factors (TFs) are well-known to regulate various developmental processes of plants, and several TFs have been reported to be specifically related to plant sex determination and sex differentiation. In the present study, among the DEGs identified between the SFs and PFs of mulberry, a total of 332 TFs were found (summarized in Supplementary Table S7). It has been proven that MADS-box TFs are critical for proper floral development and fruit production of some plants, including mulberry (Yao et al., 2001; Bartlett, 2017; Shang et al., 2017). In this DEGs’ database, most of the MADS-box TFs had an obvious upward trend in the PFs of mulberry, and so they might be associated with floral development and fruit production of mulberry PFs. In research on other plants, *AGAMOUS-LIKE 9 (AGL9)* was over-expressed in the female flower of *J. curcas*, while *AGAMOUS-LIKE 24 (AGL24)* was highly expressed in male buds of *M. glyptostroboides*, with an overexpression of *AGL24* inducing early flowering of *A. thaliana* (Zhao et al., 2015; Hui et al., 2017). In the present study, two genes (*Malba05G008459* and *Malba05G008463*) which are both homologous to *pMADS 2* (one MADS-box gene), were over-expressed in the mulberry SFs, not unlike its upregulation in male floral buds of *D. kaki* (Li J. et al., 2019).

As a kind of nucleic acid binding TFs, WRKY TFs are the key regulatory components of plant development. Most of the WRKY TFs in our DEGs’ database were found to be upregulated in the PFs, and this result suggests that WRKY TFs of DEGsmaybe participated predominantly in ovary development and fruit fertilization. For example, overexpression of WRKY75 accelerates flowering by interacting with DELLA proteins in *A. thaliana* (Zhang et al., 2018), hence, the three WRKY75 over-expressed in the SFs of mulberry could be related to the early flowering of SFs. In this study, four *LRR receptor-like serine/threonine-protein kinase* genes (LRR-RLKs) that belong to the LRR receptor kinase family are downregulated in the SFs of mulberry. LRR-RLKs act upon the substrates which control floral organ abscission (Cho et al., 2008), and some of them were found to be involved in the floral growth and development of *S. japonicus* (Li J. et al., 2019). In *Carica papaya*, two *LRR-RLK* genes were upregulated in its female buds and may be involved in that plant’s sex differentiation (Zerpa-Catanho et al., 2019). The C2H2 TFs have been previously documented to play important roles in various processes during plant development, including sex determination. In this study of mulberry, thirty genes for C2H2 TF showed male-biased expression and seven genes for C2H2 TF exhibited female-biased expression patterns, results that contrasted with most C2H2 family genes being primarily female-biased in *A. officinalis* (Li et al., 2017). Of the male-biased C2H2 TF genes in this study, the *zinc finger proteins ZAT4* and *zinc finger proteins ZAT10* were reportedly downregulated in female flower buds compared to hermaphrodites flowers of *Silene vulgaris* which suggests their relevance for conferring to play important role in male fertility (Krüger et al., 2020). Finally, in *A. thaliana*, two C2H2 TFs participated in the development of female structures in the carpel (Sagasser et al., 2002; Crawford et al., 2007).

## Conclusion

Multiomics technologies are now widely used to characterize mechanisms of physiological processes in organisms. Here, integrative transcriptomics and metabolomics analyses were used to examine the regulatory mechanisms of sex differentiation of mulberry flowers. The changes in the MAPK pathway, flavonoid biosynthesis, galactose metabolism, plant–pathogen interaction, and starch and sucrose metabolism between SFs and PFs imply their important roles in the sex differentiation of mulberry flowers. The functional annotation of high-throughput transcriptomics and metabolomics data generated in this study can serve as a genetic and metabolomics resource for the development of tree improvement strategies for mulberry and other similar economically valuable trees.

## Data Availability

The datasets presented in this study can be found in online repositories. The names of the repository/repositories and accession number(s) can be found at: https://www.ncbi.nlm.nih.gov/, PRJNA755850.
