# Supplementary material for: Integrative Analyses of Transcriptomics and Metabolomics in Sex Differentiation of Mulberry Flowers
Source: Front Mol Biosci. 2022 May 5;9:881090. doi: 10.3389/fmolb.2022.881090 (PMC9117626; doi:10.3389/fmolb.2022.881090)
Supplement: Supplementary file 3 [file Table1.DOCX]

Table S1 The primers sequences used for qRT-PCR analysis on selected DEGs

| Genes | Forward primer (5’-3’) | Reverse primer(5’-3’) |
| --- | --- | --- |
| Malba09G014845 | GGCTGTGGTTGCATTAGTC | TCGTCCTCTCTGTTTGGTT |
| Malba11G017549 | ATATGGAGTTCAGATCGAGAG | ATGGTAAGTGATGACAGAGTG |
| Malba03G003945 | CGGCACTTGATGACCAACCTC | CGTCCTCTTGCTCCTTCGCTT |
| Malba01G000467 | TGTTCTCCGACAAGCATTCTC | ACCCAACTCTGGTCCCCACTA |
| Malba05G006734 | CACCGATAACACGAACGCCGC | CAATTTTCCTCATTAACAGCC |
| Malba09G014185 | CATCATAAGCGTCTCTTCGTC | CTTTCTCTTTTTGGATCTCGG |
| Malba04G005485 | GATAGAGAACAAAGAGGGGCT | GACATTGGAGATAGTGGAGGT |
| Malba05G007811 | CAACTGTTTACTTTTGCTGAT | ATTCTTGTCCATTTTTTCCTC |
| Malba07G012074 | AGGAACTAGAAAAAATGGTGG | TAACTTGAAATGTGCGAGAGG |
| Malba14G022196 | AGGAGAAAGCACAGCAACCAC | CTCAACTCTAGAAAAGACACG |
| Morus024083（*ribosomal protein* gene） | GGCTATGTGATTTACCGTGTT | TTGGTCCAGTATGAGTTGAGAA |
